# A high molar activity ^18^F-labeled TAK-875 derivative for PET imaging of pancreatic *β*-cells

**DOI:** 10.1186/s41181-018-0051-2

**Published:** 2018-12-20

**Authors:** Mark H. Dornan, Daniil Petrenyov, José-Mathieu Simard, Antonio Aliaga, Guoming Xiong, Julien Ghislain, Barry Bedell, Vincent Poitout, Jean N. DaSilva

**Affiliations:** 1 Centre de Recherche du Centre Hospitalier de l’Université de Montréal, 900 rue St. Denis, Montréal, Québec, H2X 0A9 Canada; 2 Département de radiologie, radio-oncologie et médecine nucléaire, Université de Montréal, 2900 boulevard Edouard-Montpetit, Montréal, Québec, H3T 1A4 Canada; 3 Centre for Translation Biology, Research Institute of the McGill University Health Centre, 1001 boulevard Décarie-Block E, Montréal, Québec, H4A 3J1 Canada; 4 Département de médecine, Université de Montréal, 2900 boulevard Edouard-Montpetit, Montréal, Québec, H3T 1A4 Canada

**Keywords:** Pancreatic Beta-cell imaging, Diabetes, Free fatty acid receptor-1, Lipophilic analogs

## Abstract

**Background:**

The free-fatty acid receptor-1 (FFA-1) is expressed by *β*-cells and is a promising target for molecular imaging of functional *β*-cell mass. Recently, the ((3-[^18^F]fluoropropyl)sulfonyl)propoxy-derivative of the high-affinity FFA-1 agonist TAK-875 (**[**^**18**^**F]7**) was reported. Here we describe the preparation of this tracer in high molar activity using a purification method permitting separation of **[**^**18**^**F]7** from a structurally-related by-product and evaluation of the tracer in rats as a potential FFA-1 PET imaging agent.

**Results:**

The radiotracer was produced by nucleophilic radio-fluorination of the tosylate precursor and deprotection of the methyl ester. Semi-preparative HPLC with a C18 column revealed that **[**^**18**^**F]7** co-eluted with a non-radioactive impurity. Mass spectrometry identified the impurity as the alkene-containing elimination by-product. A pentafluorophenyl-functionalized HPLC column was found to separate the two compounds and allowed for purification of **[**^**18**^**F]7** in high molar activity. A strong anion-exchange resin was used to reformulate **[**^**18**^**F]7** in high concentration. Starting from 96 to 311 GBq of [^18^F]fluoride, 3.8–15.4 GBq of pure **[**^**18**^**F]7** (end of synthesis (EOS)) was prepared (RCY 8.3% ± 1.1% decay-corrected, *n* = 4) in high molar activity (166–767 GBq/μmol at EOS). This PET agent was evaluated in rats using dynamic microPET/CT imaging, ex vivo biodistribution, and radio-metabolite studies. MicroPET/CT exhibited high uptake of the tracer in the abdominal area. There was no measurable decrease of the PET signal in the pancreatic area in rats pre-treated with saturating doses (30 mg/kg) of TAK-875. Biodistribution studies corroborated the microPET/CT results. Radiometabolism analyses revealed high compound stability with only the parent molecule detected in the pancreas.

**Conclusions:**

Analysis of the crude reaction mixture and identification of the elimination by-product allowed for the development of a fully automated process to prepare the TAK-875-derived PET agent **[**^**18**^**F]7** in high purity and high molar activity. Even though the radiotracer exhibited high in vivo stability, microPET/CT and biodistribution results confirmed recent reports demonstrating that lipophilic analogs of TAK-875 display a high degree of non-specific binding, masking any specific binding to FFA-1 in pancreatic *β*-cells. Future development of TAK-875-derived PET tracers should focus on reducing non-specific binding in the pancreatic tissue.

## Background

Type II diabetes mellitus (T2DM) is the predominant form of diabetes. The disease is characterized by insulin resistance and, after an initial compensatory over-production of insulin by *β*-cells, a loss of *β*-cell functional mass (Kahn 2003). Current understanding of *β*-cell dysfunction in T2DM has largely come from post-mortem autopsy data. There have been considerable efforts made towards developing a non-invasive imaging method to monitor *β*-cell function for both research and clinical studies (Andralojc et al. 2012). Such research would allow better understanding of *β*-cell mass biology including the pathological mechanisms that lead to depletion during disease progression and could be used in longitudinal studies in the pre-clinical and clinical evaluation of new therapies.

Insulin-secreting *β*-cells are localized in small (20 to 600 μM in diameter) clusters of endocrine cells called the islets of Langerhans, which are dispersed throughout the pancreas and account for only 1 to 2% of the total pancreatic mass (Ionescu-Tirgoviste et al., 2015). The size of this anatomical structure and its placement deep within the body restricts potential imaging modalities to those with high sensitivity, spatial resolution, and penetration depth. Given this, nuclear molecular imaging using techniques such as positron emission tomography (PET) or single photon emission computed tomography (SPECT) hold the most promise.

The free fatty acid receptor-1 (FFA-1) is a G-coupled protein receptor that is highly expressed in human and rodent *β*-cells (Itoh et al. 2003; Briscoe et al. 2002). FFA-1 has attracted significant attention as a potential therapeutic target to promote glucose-stimulated insulin secretion (Mancini and Poitout 2015). The FFA-1 partial agonist TAK-875 was developed as a potent (K_D_ = 4.8 nM and 6.3 nM for human and rat FFA-1, respectively) and selective therapeutic agent that reached phase III clinical trials before development was halted due to liver toxicity (Negoro et al. 2010; Otieno et al. 2017). Structure-activity relationship and TAK-875/FFA-1 co-crystal structure studies suggest that the 3-(methylsulfonyl)propoxy-moiety of TAK-875 could be modified to allow for radionuclide incorporation without significantly impacting target binding (Negoro et al. 2012; Srivastava et al. 2014).

Bertrand and coworkers recently reported the synthesis of TAK-875-derived fluorescent probes for *β*-cell imaging (Bertrand et al. 2016a). These new probes were used to track specific labeling of cells overexpressing the FFA-1 receptor. Later in 2016, while we were working on the same tracer, Bertrand and coworkers published the radiosynthesis of the ((3-[^18^F]fluoropropyl)sulfonyl)propoxy-derivative of TAK-875 (**[**^**18**^**F]7)** in 16.7% ± 5.7% radiochemical yield (RCY) in low molar activity (≥0.6 GBq/μmol) (Bertrand et al. 2016b). We report here a fully automated process to produce **[**^**18**^**F]7**, including optimized HPLC purification and reformulation conditions allowing for removal of the elimination by-product to obtain a high purity, high molar activity, and concentrated formulation of **[**^**18**^**F]7** for in vivo evaluation in rats.

## Methods

### General

All commercially available materials were used without further purification unless otherwise noted. The synthesis of **1** was completed as previously described (Negoro et al. 2012). Flash chromatography was carried out with 40–60 μm silica gel. ^1^H and ^13^C NMR spectra were acquired with a 300 MHz spectrometer (Bruker) at ambient temperature. Spectral data is reported in parts per million using residual solvent as a reference. High resolution and accurate mass (HR/AM) measurements were performed in positive and negative ion mode by flow injection analysis into the Thermo Scientific Q-Exactive Plus Orbitrap Mass Spectrometer (San Jose, CA, USA) interfaced with a heated electrospray ion source.

An automated synthesis platform (IBA Synthera®) containing two synthesis modules and one semi-preparative radio-HPLC module was used for automated radiotracer production. The production was performed with the Synthera Nucleophilic Integrated Fluidic Processor research and development cassettes (Huayi Isotopes). The cassettes were cleaned with water, ethanol, and dried with nitrogen gas before each usage. Sep-Pak C18 Plus Light cartridges (130 mg, Waters) were pre-conditioned with 10 mL of ethanol followed by 20 mL of deionized water. Sep-Pak QMA Plus Light cartridges (130 mg, Waters) were pre-conditioned by passing through 10 mL of sodium bicarbonate solution (8.4%), followed by 10 mL of deionized water, and drying with helium gas. Semi-preparative HPLC purification was carried out with a Phenomenex Luna PFP (2) column (250 × 10 mm, 5 μm, flow rate, 8 mL/minute, 50% CH_3_CN/H_2_O + 0.1% TFA). A Phenomenex Luna C18 (2) column (250 × 4.6 mm, 10 μm, flow rate, 2 mL/minute, 35% CH_3_CN/H_2_O + 0.1% TFA) was utilized for analytical HPLC with a Waters HPLC equipped with a Raytest Gabi Star radioactivity detector. Residual solvent analyses were conducted using an Agilent 7890B gas chromatograph (headspace sampler).

### Chemistry

**3-(3-(tert-Butyl-dimethyl-silanyloxy)-propylsulfanyl)-propan-1-ol (2).** The product **2** was prepared using a previously published method (Wilke et al. 2014). 3,3′-Thiodipropanol (870 mg, 5.8 mmol) was added to acetonitrile (12 mL) and hexanes (48 mL). Triethylamine (0.97 mL, 6.95 mmol) and tert-butyldimethylsilyl chloride (873 mg, 5.8 mmol) were added and the biphasic mixture was stirred vigorously at room temperature for 8 h. The reaction was quenched with a solution of saturated NH_4_Cl and extracted with EtOAc. The combined organic fractions were washed with brine, dried over MgSO_4,_ filtered, and concentrated. Compound **2** (903 mg, 3.41 mmol, 59%) was purified by silica column chromatography (10% to 30% EtOAc in hexanes). TLC R*f* = 0.44 in 30% EtOAc/hexanes. ^1^H NMR (300 MHz, CDCl_3_) δ 3.73 (t, *J* = 6.1 Hz, 2H), 3.67 (t, *J* = 6.1 Hz, 2H), 2.69–2.52 (m, 4H), 1.97 (s, 1H), 1.89–1.71 (m, 4H), 0.87 (s, 9H), 0.04 (s, 6H). ^13^C NMR (75 MHz, CDCl_3_) δ 61.9, 61.7, 32.7, 32.0, 28.9, 28.6, 26.0, 18.4, − 5.2. HRMS (ESI): Exact mass calcd for C_12_H_29_O_2_SSi [M + H]^+^: 265.1652. Found: 265.1652.

**(S)-methyl-2-(6-((4′-(3-((3-((*tert*-butyldimethylsilyl)oxy)-propyl)thio)propoxy)-2′,6′-dimethyl-[1,1′-biphenyl]3-yl)methoxy)-2,3-dihydrobenzofuran-3-yl)acetate (3). ** Compound **1** (950 mg, 2.27 mmol, 1.0 equiv.), **2** (600 mg, 2.27 mmol, 1.0 equiv.), and P(*n*-Bu_3_) (0.906 mL, 3.63 mmol, 1.6 equiv.) were dissolved in toluene (45 mL). 1,1′-(Azodicarbonyl)dipiperidine (0.916 g, 3.63 mmol, 1.6 equiv.) was added portion wise. The reaction was stirred for 20 h under N_2_ atmosphere at room temperature. The reaction mixture was diluted with H_2_O and extracted with EtOAc. The desired compound **3** (1.05 g, 1.585 mmol, 70%) was purified by silica column chromatography (2% to 8% EtOAc in hexanes). TLC R*f* = 0.35 in 15% EtOAc/hexanes. ^1^H NMR (300 MHz, CDCl_3_) δ 7.47–7.34 (m, 2H), 7.17 (s, 1H), 7.08 (d, *J* = 6.9 Hz, 1H), 7.02 (d, *J* = 8.0 Hz, 1H), 6.66 (s, 2H), 6.54–6.42 (m, 2H), 5.06 (s, 2H), 4.75 (t, *J* = 9.0 Hz, 1H), 4.26 (dd, *J* = 9.1, 6.1 Hz, 1H), 4.08 (t, *J* = 6.0 Hz, 2H), 3.87–3.75 (m, 1H), 3.75–3.64 (m, 5H), 2.81–2.49 (m, 6H), 2.13–1.95 (m, 8H), 1.88–1.75 (m, 2H), 0.91 (s, 9H), 0.07 (s, 6H). ^13^C NMR (75 MHz, CDCl_3_) δ 172.7, 161.4, 160.3, 158.0, 141.5, 137.7, 137.4, 134.6, 129.5, 129.0, 128.9, 125.8, 124.6, 121.8, 113.5, 107.6, 97.8, 77.9, 70.6, 66.4, 61.9, 52.1, 39.8, 38.1, 33.0, 29.7, 29.0, 28.9, 26.3, 21.4, 18.6, − 5.0. Exact mass calcd for C_38_H_53_O_6_SSi [M + H]^+^: 665.3327. Found: 665.3326.

**(S)-methyl-2-(6-((4′-(3-((3-hydroxypropyl)sulfonyl)propoxy)-2′,6′-dimethyl-[1,1′-biphenyl]3-yl)methoxy)-2,3-dihydrobenzofuran-3-yl)acetate (4).** The compound **3** (275 mg, 0.413 mmol, 1.0 equiv.) was dissolved in CH_3_OH (5 mL) and THF (5 mL) and the mixture was cooled to 0 °C. Oxone (254 mg, 0.827 mmol, 2.0 equiv.) dissolved in H_2_O (5 mL) was added to the mixture dropwise. The reaction mixture was stirred from 0 °C to room temperature for 18 h. The reaction mixture was concentrated to remove the organic solvent and the aqueous residue was diluted with H_2_O. The mixture was extracted with EtOAc, washed with brine, dried over MgSO_4_, filtered, and concentrated. Product **4** (223 mg, 0.383 mmol, 93%) was purified by silica column chromatography (50% to 90% EtOAc in hexanes). TLC R*f* = 0.43 in 90% EtOAc/hexanes. ^1^H NMR (300 MHz, CDCl_3_) δ 7.47–7.33 (m, 2H), 7.16 (s, 1H), 7.07 (d, *J* = 7.1 Hz, 1H), 7.01 (d, *J* = 8.0 Hz, 1H), 6.64 (s, 2H), 6.51–6.43 (m, 2H), 5.05 (s, 2H), 4.74 (t, *J* = 9.0 Hz, 1H), 4.26 (dd, *J* = 9.2, 6.1 Hz, 1H), 4.12 (t, *J* = 5.7 Hz, 2H), 3.88–3.74 (m, 3H), 3.71 (s, 3H), 3.32–3.12 (m, 4H), 2.74 (dd, *J* = 16.4, 5.5 Hz, 1H), 2.55 (dd, *J* = 16.4, 9.2 Hz, 1H), 2.42–2.28 (m, 2H), 2.21–2.07 (m, 2H), 1.99 (s, 6H), 1.72 (s, 1H). ^13^C NMR (75 MHz, CDCl_3_) δ 172.7, 161.4, 160.2, 157.4, 141.2, 137.9, 137.5, 135.1, 129.4, 129.0, 128.9, 125.9, 124.6, 121.8, 113.5, 107.6, 97.8, 77.9, 70.6, 65.7, 60.9, 52.1, 50.4, 50.3, 39.8, 38.1, 25.3, 22.7, 21.4. HRMS (ESI): Exact mass calcd for C_32_H_39_O_8_S [M + H]^+^: 583.2360. Found: 583.2366.

**(S)-methyl-2-(6-((2′,6′-dimethyl-4′-(3-((3-(tosyloxy)propyl)sulfonyl)propoxy)-[1,1′-biphenyl] 3-yl)methoxy)-2,3-dihydrobenzofuran-3-yl)acetate (5). ** Product **5** was prepared as described previously (Bertrand et al. 2016b). *p*-Toluenesulfonyl chloride (196 mg, 1.03 mmol, 1.5 equiv.) dissolved in 1.9 mL of CH_2_Cl_2_ was added dropwise to a solution of **4** (400 mg, 0.686 mmol, 1.0 equiv.) dissolved in toluene (4.7 mL) and CH_2_Cl_2_ (4.7 mL), in the presence of triethylamine (0.143 mL, 1.03 mmol, 1.5 equiv.) and *N*,*N*,*N′*,*N′*-tetramethyl-1,6-hexanediamine (15 μL, 0.0686 mmol, 0.1 equiv.). The reaction was stirred for 18 h at room temperature. The mixture was quenched with H_2_O, extracted with EtOAc, washed with brine, dried over MgSO_4_, filtered and concentrated. The product **5** (463 mg, 0.628 mmol, 92%) was purified by silica column chromatography (30% to 50% EtOAc in hexanes). TLC R*f* = 0.40 in 50% EtOAc/hexanes. ^1^H NMR (300 MHz, CDCl_3_) δ 7.79 (d, *J* = 8.3 Hz, 2H), 7.46–7.32 (m, 4H), 7.16 (s, 1H), 7.07 (dt, *J* = 7.1, 1.6 Hz, 1H), 7.02 (d, *J* = 8.0 Hz, 1H), 6.64 (s, 2H), 6.52–6.43 (m, 2H), 5.06 (s, 2H), 4.75 (t, *J* = 9.0 Hz, 1H), 4.26 (dd, *J* = 9.2, 6.1 Hz, 1H), 4.19 (t, *J* = 5.9 Hz, 2H), 4.11 (t, *J* = 5.7 Hz, 2H), 3.87–3.74 (m, 1H), 3.71 (s, 3H), 3.28–3.16 (m, 2H), 3.16–3.05 (m, 2H), 2.75 (dd, *J* = 16.4, 5.5 Hz, 1H), 2.55 (dd, *J* = 16.4, 9.2 Hz, 1H), 2.46 (s, 3H), 2.38–2.19 (m, 4H), 1.99 (s, 6H). ^13^C NMR (75 MHz, CDCl_3_) δ 172.6, 161.4, 160.2, 157.4, 145.6, 141.2, 137.9, 137.5, 135.2, 132.9, 130.4, 129.4, 129.0, 128.9, 128.3, 125.9, 124.6, 121.8, 113.5, 107.6, 97.8, 77.9, 70.6, 68.3, 65.6, 52.1, 50.7, 49.4, 39.8, 38.1, 22.6, 22.2, 22.0, 21.4. Exact mass calcd for C_39_H_45_O_10_S_2_ [M + H]^+^: 737.2449. Found: 737.2442.

**(S)-methyl-2-(6-((2′,6′-dimethyl-4′-(3-((3-fluoropropyl)sulfonyl)propoxy)-[1,1′-biphenyl] 3-yl)methoxy)-2,3-dihydrobenzofuran-3-yl)acetate (6).** Product **6** was prepared as described previously (Bertrand et al. 2016b). 50% Deoxo-fluor (bis(2-methoxyethyl)aminosulfur trifluoride) in THF (37 mg, 0.166 mmol, 1.65 equiv.) was added to **4** (59 mg, 0.101 mmol, 1.0 equiv.) in CH_2_Cl_2_ at 0 °C. The reaction was allowed to react from 0 °C to room temperature for 18 h. The reaction mixture was diluted with H_2_O, extracted with EtOAc, dried over Na_2_SO_4_, filtered and concentrated. The desired product **6** (31 mg, 0.053 mmol, 53%) was purified by silica column chromatography (20% to 50% EtOAc in hexanes). TLC R*f* = 0.51 in 50% EtOAc/hexanes. ^1^H NMR (300 MHz, CDCl_3_) δ 7.46–7.34 (m, 2H), 7.16 (s, 1H), 7.07 (d, *J* = 7.0 Hz, 1H), 7.02 (d, *J* = 8.0 Hz, 1H), 6.64 (s, 2H), 6.52–6.44 (m, 2H), 5.05 (s, 2H), 4.75 (t, *J* = 9.0 Hz, 1H), 4.68 (t, *J* = 5.6 Hz, 1H), 4.53 (t, *J* = 5.6 Hz, 1H), 4.26 (dd, *J* = 9.2, 6.1 Hz, 1H), 4.13 (t, *J* = 5.7 Hz, 2H), 3.86–3.74 (m, 1H), 3.72 (s, 3H), 3.29–3.14 (m, 4H), 2.75 (dd, *J* = 16.4, 5.5 Hz, 1H), 2.55 (dd, *J* = 16.4, 9.2 Hz, 1H), 2.42–2.19 (m, 4H), 1.99 (s, 6H). ^13^C NMR (75 MHz, CDCl_3_) δ 172.67, 161.45, 160.25, 157.40, 141.25, 137.98, 137.49, 135.18, 129.45, 128.99, 128.89, 125.94, 124.61, 121.83, 113.52, 107.63, 97.79, 82.01 (d, *J*_*C,F*_ = 168 Hz), 77.90, 70.61, 65.70, 52.13, 50.59, 49.55 (d, *J*_*C,F*_ = 4 Hz), 39.81, 38.09, 23.57 (d, *J*_*C,F*_ = 21 Hz), 22.67, 21.45. HRMS (ESI): Exact mass calcd for C_32_H_39_O_8S_ [M + Na]^+^: 607.2136. Found: 607.2136.

**(S)-2-(6-((4′-(3-((3-fluoropropyl)sulfonyl)propoxy)-2′,6′-dimethyl-[1,1′-biphenyl]3-yl)methoxy)-2,3-dihydrobenzofuran-3-yl)acetic acid (7).** Product **7** was prepared as described previously (Bertrand et al. 2016b). NaOH_(aq)_ (1.0 M, 0.17 mL) was added to a solution of **6** (20 mg, 0.034 mmol, 1.0 equiv.) in CH_3_OH (0.3 mL) and THF (0.6 mL), and the mixture was heated to 50 °C for 1 h. The reaction mixture was concentrated and **7** (17.3 mg, 0.030 mmol, 88%) was purified by silica column chromatography (80% EtOAc in hexanes + 0.5% CH_3_CO_2_H). TLC R*f* = 0.40 in 80% EtOAc/hexanes + 0.5% CH_3_CO_2_H. ^1^H NMR (300 MHz, CDCl_3_) δ 7.47–7.33 (m, 2H), 7.16 (s, *J* = 10.1 Hz, 1H), 7.06 (t, *J* = 7.4 Hz, 2H), 6.64 (s, 2H), 6.53–6.43 (m, 2H), 5.05 (s, 2H), 4.75 (t, *J* = 9.0 Hz, 1H), 4.68 (t, *J* = 5.6 Hz, 1H), 4.53 (t, *J* = 5.6 Hz, 1H), 4.28 (dd, *J* = 9.2, 6.1 Hz, 1H), 4.12 (t, *J* = 5.7 Hz, 2H), 3.87–3.72 (m, 1H), 3.30–3.13 (m, 4H), 2.80 (dd, *J* = 16.7, 5.2 Hz, 1H), 2.60 (dd, *J* = 16.7, 9.3 Hz, 1H), 2.41–2.28 (m, 3H), 2.28–2.19 (m, 1H), 1.99 (s, 6H). ^13^C NMR (75 MHz, CDCl_3_) δ 161.43, 160.31, 157.40, 141.26, 137.98, 137.45, 135.17, 129.48, 129.00, 128.89, 125.94, 124.63, 121.58, 113.53, 107.72, 97.84, 82.0 (d, *J*_*C,F*_ = 168 Hz), 70.62, 65.70, 50.58, 49.55 (d, *J*_*C,F*_ = 4 Hz), 37.91, 23.56 (d, *J*_*C,F*_ = 21 Hz), 22.65, 21.45. Exact mass calcd for C_31_H_35_FNaO_7_S [M + Na]^+^: 593.1980. Found: 593.1984.

### Production of [^18^F]7

**Fig. 1:**
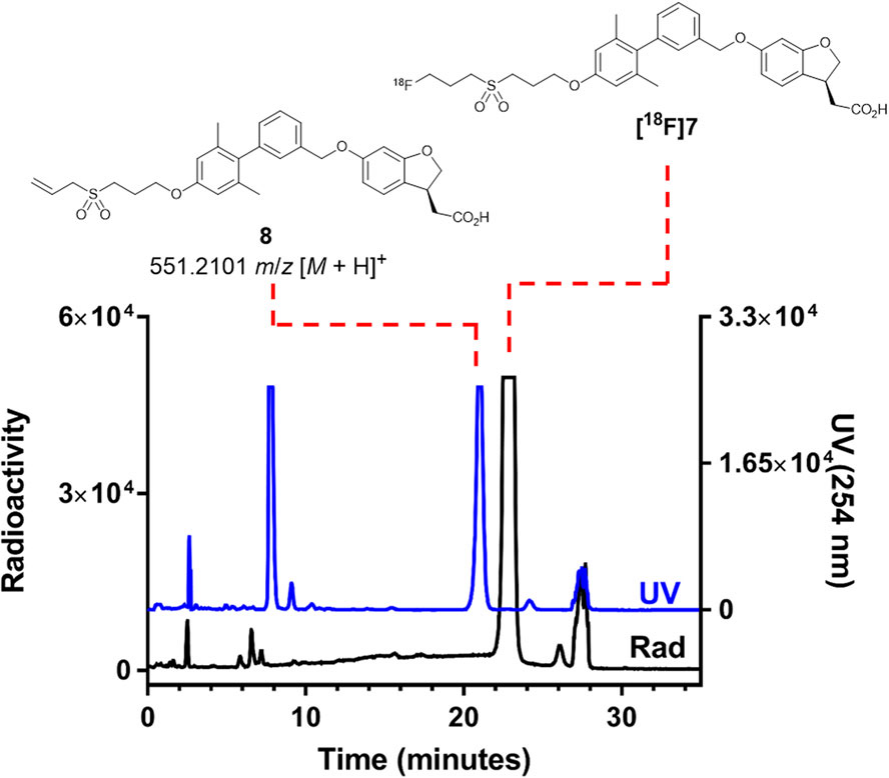
Semi-preparative HPLC chromatogram of a typical radiosynthesis of **[**^**18**^**F]7**. Resolution of **[**^**18**^**F]7** (t_R_ = 23 min) from the elimination by-product **8** (t_R_ = 21 min) was achieved using a pentafluorophenyl functionalized HPLC column. Radioactivity (black) and UV (γ = 254 nm; blue)

Using an IBA Cyclone® 18/9 cyclotron, [^18^F]fluoride was produced by the ^18^O(p,n)^18^F nuclear reaction and was transferred to the IBA Synthera® radiochemistry platform which was configured with two synthesis units and an HPLC module. The [^18^F]fluoride was trapped on a pre-conditioned Waters Sep-Pak® QMA Plus Light anion-exchange cartridge. [^18^F]Fluoride was eluted from the cartridge into the reactor with 0.6 mL of a 1:1 mixture of acetonitrile/water containing 2.2.2-cryptand (22.6 mg) and potassium carbonate (4.2 mg). Acetonitrile was added, and the mixture was azeotropically dried under vacuum and heat. A solution of the tosylate precursor **5** (5 mg) in 1 mL of acetonitrile was added and the reactor was heated to 100 °C for 2.5 min. Following removal of the solvent (vacuum), the reactor was cooled and 1 mL of a 1:1 mixture of aqueous NaOH (2.5 M)/CH_3_OH was added. After 3 min, the reaction was quenched with 10 mL of H_2_O + 0.1% TFA and transferred through a pre-conditioned Waters Sep-Pak® C18 cartridge. Acetonitrile (1.2 mL) eluted the radiotracer from the C18 cartridge into a V-vial containing H_2_O + 0.1% TFA (1.8 mL) and injected onto semi-preparative HPLC equipped with a Phenomenex Luna PFP (2) column, which enabled separation of the radiotracer from the elimination by-product **8** (Fig. 1). The fraction containing **[**^**18**^**F]7** was collected into a vessel containing aqueous NaOH (1.0 M, 30 mL) and transferred over a Waters Oasis® MAX anion-exchange resin cartridge using vacuum and N_2_ push in the second Synthera synthesis unit. Following deionized water wash (10 mL), **[**^**18**^**F]7** was eluted with 1 mL EtOH + 0.5% HCl, then filtered (0.22 μm) into a sterile multidose vial containing 9 mL of isotonic bicarbonate-buffered saline. Analytical HPLC was performed to determine the chemical and radiochemical purity. Identity of **[**^**18**^**F]7** was determined by HPLC co-elution with the non-radioactive standard **7**. The molar activity was measured by comparing the UV (254 nm) peak of **[**^**18**^**F]7** with an injection of the non-radioactive standard **7** with a comparable UV response.

### Identification of the impurity 8

The radiosynthesis and HPLC purification of **[**^**18**^**F]7** was conducted as described above using a semi-preparative Phenomenex Luna C18 column. The HPLC peak containing a co-elution of **[**^**18**^**F]7** and a non-radioactive impurity was collected. The decayed fraction was diluted in methanol and analyzed by mass spectrometry. HRMS (ESI): Exact mass calcd for C_31_H_35_O_7_S [M + H]^+^: 551.2098. Found: 551.2101.

### Animals

Male Sprague-Dawley rats (150–175 g, Charles River Laboratories, Montreal, Canada) were housed on a 12 h:12 h light/dark cycle and fed standard rat chow and water ad libitum. This study was carried out in accordance with Canadian Council on Animal Care guidelines. The protocol (protocol number IM16029JDSr) was approved by the institutional Animal Care Committees of the Centre de Recherche du Centre Hospitalier de l’Université de Montreal and the McGill University Health Centre.

### Small animal PET imaging

#### General

Small animal PET/CT scans were performed with **[**^**18**^**F]7** (7.4–14.8 MBq, 0.12–0.66 μg non-radioactive mass) in control animals (*n* = 3) on a Mediso nanoScan PET/CT (Mediso Medical Imaging Systems, Budapest, Hungary). To assess binding specificity to FFA-1 in the pancreas, additional rats (n = 3) were injected with TAK-875 (30 mg/kg, from a 20 mg/mL solution of TAK-875 in 10,5,1 saline 0.9%/H_2_O/sodium bicarbonate 8.4% + 10% DMSO) intravenously 5 min before administration of **[**^**18**^**F]7** (12.8–13.5 MBq, 0.27–0.58 μg non-radioactive mass).

#### Image acquisition

Each PET/CT session consisted of a 60 min PET emission scan, followed by a 10 min CT transmission scan. PET/CT scans were conducted under anesthesia (isoflurane 2%, oxygen 0.6 L/min) delivered by a nose cone. Temperature and heart rate were monitored throughout the procedure using the Mediso system. After the animal was placed in the scanner, with the heart positioned at the center of the field of view, emission scans were initiated immediately after a bolus injection in the tail vein. List mode data was histogrammed into 29 sequential time frames of increasing duration (6 × 10 s, 4 × 30 s, 4 × 60 s, 4 × 120 s, 5 × 180 s, 6 × 300 s frames) over 60 min. Images were reconstructed using expectation maximization (EM), ordered subset expectation maximization (OSEM), normalized, and corrected for scatter, dead time, and decay.

#### Image analysis

Images were analyzed with Amide software (version 1.0.5 for Linux). Regions of interest (ROI) were defined on reconstructed images in the pancreas, kidney, liver and muscle to obtain time-activity curves. A 3-D sphere with a radius of 2.5 mm was drawn within the left ventricle using an average image of very early frames (10–60 s) to sample the blood input function. The sphere was centred at the point where the maximum activity was found inside this region. The ROIs for pancreas, kidney, liver and muscle were drawn using an average image of early frames (1–20 min post injection where highest tissue to background contract can be obtained). For kidney and liver, the ROIs are 3-D spheres located roughly in the middle of the tissue with a radius of 5 mm (kidney) and 6 mm (liver), respectively. For pancreas, the ROI was ellipsoid shaped and was created based on the anatomy of rats as both CT and PET images could not help with distinguishing this tissue from other organs.. The radii of the ROI were 1.5 mm (sagittal), 3 mm (transverse), and 3 mm (coronal), respectively. For muscle, the ROI was a 3-D sphere located on the muscle near left shoulder of the animals with a radius of 3 mm.

### Statistical analysis

Results are presented as mean ± standard deviation. A two-tailed t-test was used to compare between two groups. *P* values < 0.05 were considered significant.

### Biodistribution studies

Biodistribution studies were performed to evaluate tissue uptake and to measure specific and non-specific binding of the radiotracer to FFA-1. Briefly, rats were anesthetized (2–2.5% isofluorane) and injected via a lateral tail vein with 7.8–12.2 MBq tracer activity. Baseline group animals (*n* = 4) received tracer alone, whereas the blocking group animals (n = 4) received an intravenous dose of the FFA-1 agonist TAK-875 (30 mg/kg) 5 min prior to tracer administration. Rats were sacrificed by decapitation at 20 min post-injection. Trunk blood was collected in heparinized tubes. The following tissues were dissected and collected into pre-weighed tubes: pancreas, kidney, spleen, fat, bone, muscle, blood, plasma. Using a gamma counter (PerkinElmer Wizard2), all tubes were counted along with a 1:100 dilution of the injected tracer solution as a standard. The percent of the injected dose per gram of tissue (%ID/g) was calculated from the decay-corrected counts and expressed as a ratio to blood.

### Radiolabeled metabolite analysis

#### General

Radiolabeled metabolite analysis studies were performed to evaluate the metabolic stability of **[**^**18**^**F]** in plasma and the pancreas. Rats were anesthetized (2–2.5% isofluorane) and injected via the lateral tail vein with 35.2–73.3 MBq of **[**^**18**^**F]7**. At 60-min post-injection, the animals were sacrificed by decapitation. Trunk blood was collected into heparinized tubes and the pancreas was dissected.

#### Sample preparation

Blood was centrifuged (4000 x g, 5 min, 4 °C) to obtain plasma. Urea (1 g) was dissolved in the plasma (1 mL) and the sample was filtered (0.22 μm) and injected onto the column-switching HPLC. Pancreas samples were suspended in 80:20 EtOH/H_2_O, homogenized using a polytron and centrifuged (14.8 rpm, 20 min, 4 °C). The supernatant was collected and dried by rotary evaporation. The residue was reconstituted in 1 mL of 1% CH_3_CN/H_2_O containing 0.4 g of urea to reduce protein binding, filtered (0.22 μm), and injected onto the column-switching HPLC. Most of the radioactivity from the pancreas (and plasma) was present in the injected sample.

#### Column-switching HPLC

A modification of the column-switching HPLC procedure was used (Kenk et al. 2008) to analyse the radioactive metabolites from plasma and pancreas samples. Eluted solvents were analyzed via two detectors in series: a UV absorbance detector (Waters 2489) and a radiation detector (Raytest Gabi Star), which were connected to a chromatography data integration system (PeakSimple, using PeakSimple data analysis software, version 4.44). Using mobile solvent A (1% CH_3_CN/H_2_O + 0.1% TFA, 1 or 2 mL/minute), samples were loaded onto an in-line refillable capture column (hand-packed with 20 mg of Oasis HLB polymeric reverse phase sorbent) fitted with 2.5 μm frits. The elution of biological macromolecules was monitored by UV absorbance (254 nm) and radioactivity. When the UV signal returned to baseline, the solvent flow was switched to elute the capture column loaded sample onto the analytical column (Phenomenex Luna C18, 250 × 4.6 mm, 10 μm) using mobile Solvent B (65% CH_3_CN/H_2_O + 0.1% TFA, 2 mL/minute flow rate). Retention times were calculated from the time of switch. Radioactivity data are expressed as the percentage of the total radioactivity signal. For standards, samples of blood and pancreas from control rats were spiked with **[**^**18**^**F]7** and processed as described above for each formulation tested. Use of such controls allowed for measurement of the proportion of radiotracer retained and not-retained by the capture column during loading. If radioactivity was eluted during loading of the capture column, this activity will be identified as authentic **[**^**18**^**F]7** and not as a hydrophilic labeled metabolite.

## Results

### Chemistry and radiochemistry

**Scheme 1:**
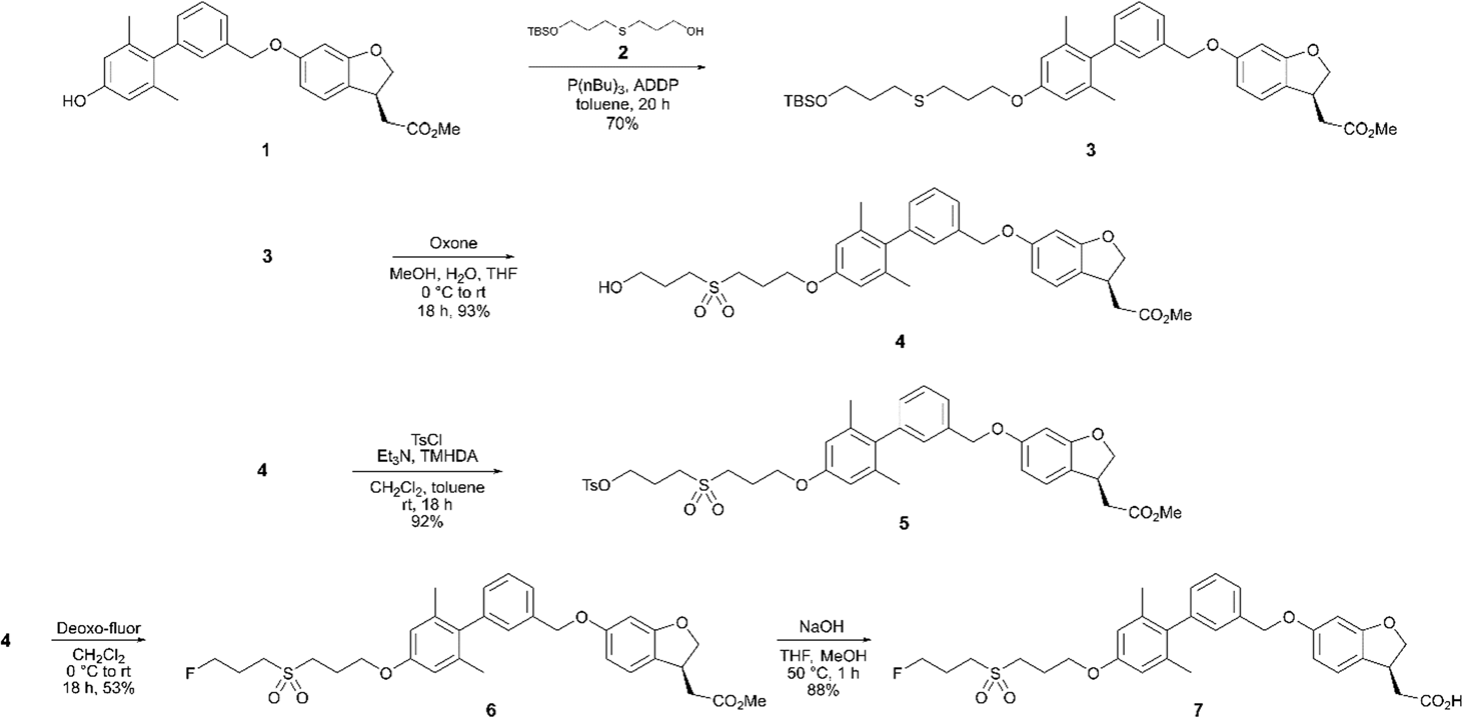
Synthesis of the radiochemical precursor **5** and the non-radioactive standard **7**

The monoprotected diol **2** was synthesized following a literature procedure (Wilke et al. 2014). A Mitsunobu coupling reaction was used to synthesize **3** in 70% yield. Treatment of **3** with Oxone oxidized the thioether to a sulfone and deprotected the terminal alcohol protecting group to afford **4** in 93% yield (Scheme 1). Tosylation of **4** provided the radiochemical precursor **5** in 92% yield. To produce the non-radioactive standard **7**, the compound **4** was treated with Deoxo-Fluor (bis(2-methoxyethyl)aminosulfur trifluoride) followed by base-mediated ester hydrolysis, as described before (Bertrand et al. 2016b).

**Scheme 2:**
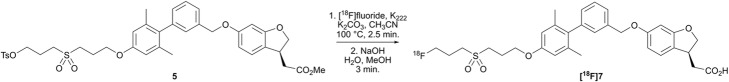
Radiosynthesis of **[**^**18**^**F]7**

The radiosynthesis of **[**^**18**^**F]7** was performed with an automated one-pot, two-step procedure. [^18^F]fluoride nucleophilic displacement of the tosylate leaving group was followed by base-mediated hydrolysis of the methyl ester (Scheme 2). The quenched reaction mixture was purified by C18 SPE cartridge followed by semi-preparative HPLC (Fig. 1). The peak corresponding to **[**^**18**^**F]7** was collected into a vessel containing aqueous NaOH, loaded onto a weak anionic exchange resin, then eluted with acidified ethanol into isotonic bicarbonate-buffered saline. This procedure allowed for rapid reformulation in high concentration. Starting from 96 to 311 GBq of [^18^F]fluoride, 3.8–15.4 GBq of [^18^F]7 (at EOS) was prepared (decay-corrected RCY 8.3% ± 1.1%, *n* = 4) in 75–89 min. The molar activity ranged from 166 to 767 GBq/μmol at EOS.

### MicroPET imaging

**Fig. 2:**
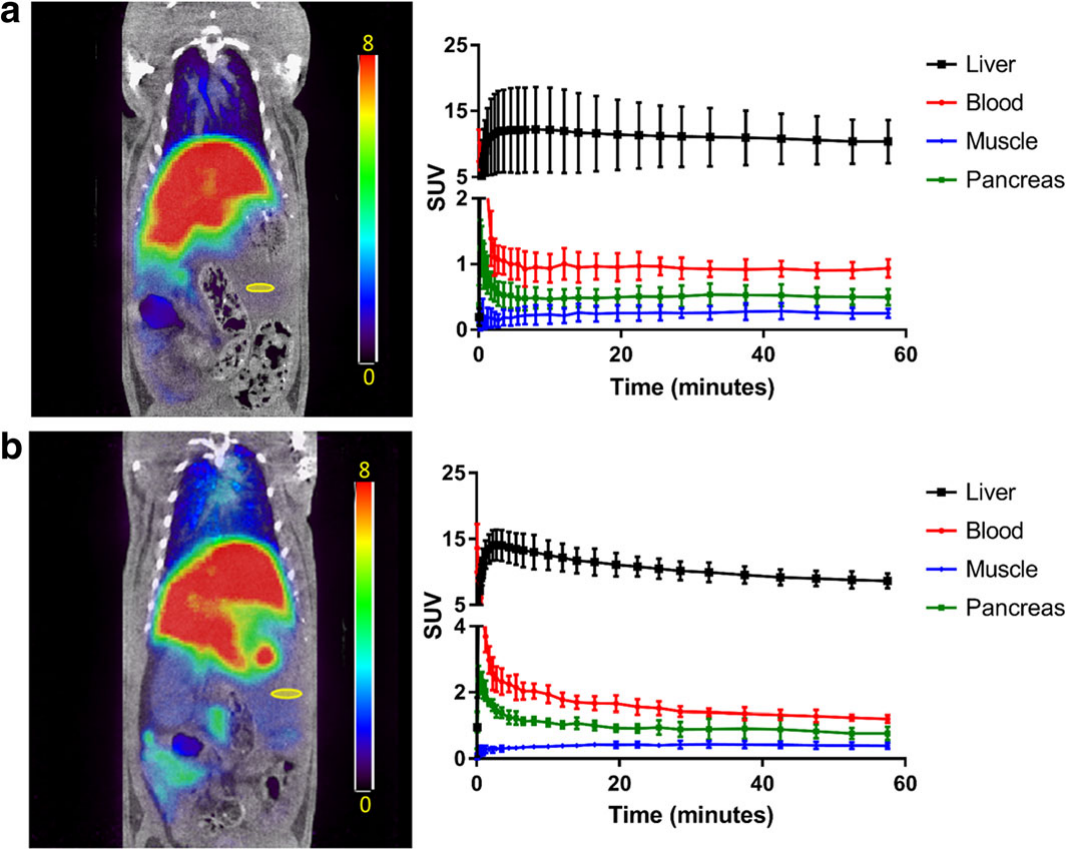
Representative microPET images (SUV, sum of all frames; pancreas ROI shown in yellow) with tissue time-activity curves in rats following injection of (**a**) **[**^**18**^**F]7** and (**b**) **[**^**18**^**F]7** with a pre-injection of TAK-875 (30 mg/kg). Time-activity curve data are presented as average SUV ± standard deviation (*n* = 3)

Preliminary microPET/CT scans performed with **[**^**18**^**F]7** exhibited the highest radiotracer concentration in the liver (Fig. 2). At 10 min post-injection, control group tissue-to-blood ratios for the liver, kidney and pancreas were 12.34 ± 3.34, 0.62 ± 0.07, and 0.49 ± 0.05, respectively (*n* = 3). In the TAK-875 pre-treated group, tissue-to-blood ratios in the liver, kidney and pancreas were 6.50 ± 0.96, 0.66 ± 0.04, and 0.57 ± 0.09, respectively (n = 3).

### Ex vivo biodistribution

**Fig. 3:**
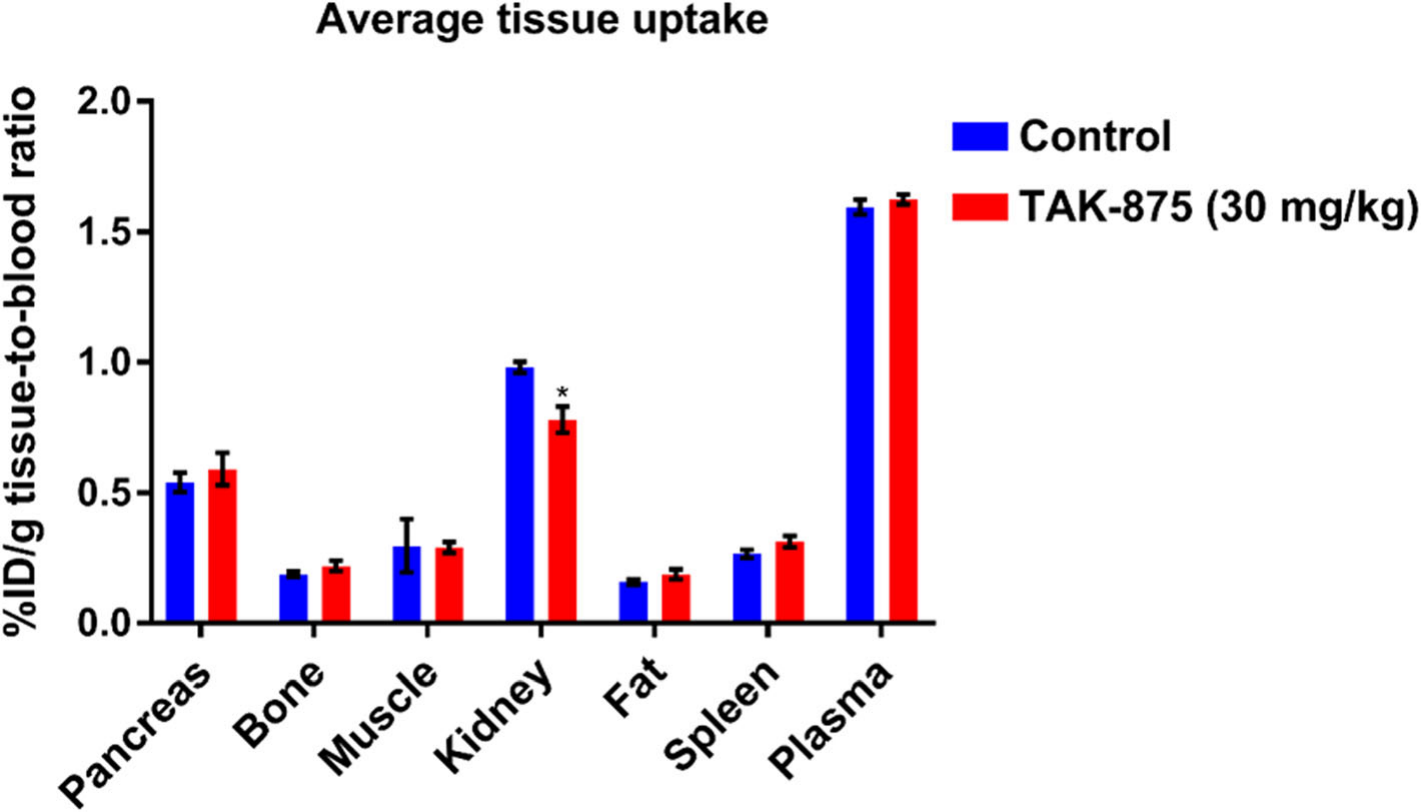
**[**^**18**^**F]7** relative tissue uptake assessed by ex vivo biodistribution in controls (*n* = 4; blue), and in rats pre-injected with 30 mg/kg TAK-875 (n = 4; red). Ratios are expressed as %ID/g ratio to blood ± SD. * *p* < 0.05 vs. control

The average % ID/g tissue-to-blood ratios for the control and blocked groups are presented in Fig. 3. In the control group, the highest average activity concentrations were found in the plasma, kidney, and pancreas (average tissue-to-blood ratios 1.60 ± 0.03, 0.98 ± 0.02, and 0.54 ± 0.04, respectively; n = 3). Lower activity concentrations were detected in the muscle, spleen, bone, and fat tissue samples (average tissue-to-blood ratios 0.30 ± 0.1, 0.27 ± 0.02, 0.19 ± 0.01, and 0.16 ± 0.01, respectively; n = 3). Blocking the FFA-1 receptor did not result in any significant difference in the tissue average activity concentrations except for the kidney, which was reduced by 20% (*P* < 0.05).

### Radiolabeled metabolites analysis

**Fig. 4:**
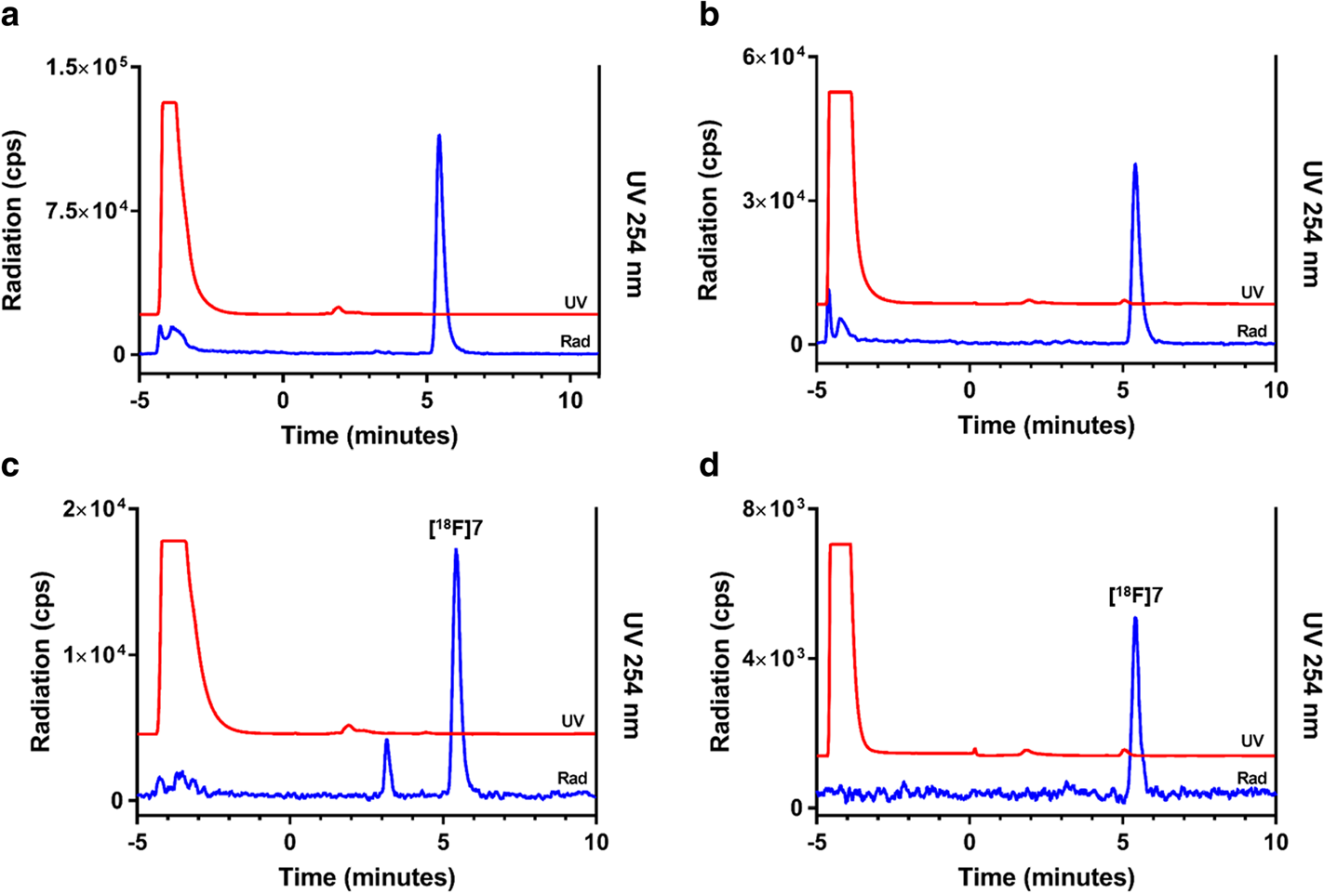
Representative column-switching HPLC radio-metabolite analysis of controls and experimental samples. Column-switching from capture-column loading to elution of the capture column onto the analytical column starts at *t* = 0 min. Radioactivity (blue) and UV (254 nm, red) spectra are shown overlaid. (**a**) Control sample plasma spiked with **[**^**18**^**F]7**; (**b**) control sample pancreas spiked with **[**^**18**^**F]7**; (**c**) 60-min post-injection in plasma; (**d**) 60-min post-injection in the pancreas

Column-switching HPLC analysis of the control plasma and pancreas samples spiked with authentic **[**^**18**^**F]7** revealed only one peak, corresponding to unchanged tracer (R_t_ = 5.5 min) (Fig. 4a-b). This demonstrated that the tissue processing method did not degrade or affect the radiotracer. No other peaks were detected in the control samples.

Sixty minutes following intravenous administration of **[**^**18**^**F]7** in rats (*n* = 4), one labeled hydrophilic metabolite was observed in rat plasma after the switch (R_t_ = 3.1 min) on analytical HPLC, while 88% ± 4% of the total radioactivity was attributed to unchanged radiotracer (Fig. 4c). In the pancreas homogenate, only the unchanged radiotracer was detected (n = 4) (Fig. 4d).

## Discussion

A novel automated method was developed to produce the ((3-[^18^F]fluoropropyl)sulfonyl)propoxy-derivative of TAK-875 (**[**^**18**^**F]7**) in high molar activity and purity, allowing for in vivo evaluation in rats as a potential FFA-1 probe for *β*-cell PET imaging. The tosylate precursor **5** and the non-radioactive standard **7** were synthesized starting from the aromatic alcohol **1**. The thioether intermediate **3** was produced by coupling the mono-protected diol **2** with the aromatic alcohol **1** using Mitsunobu-coupling conditions. Removal of the silyl protecting group and oxidation of the thioether was achieved in a single step by treatment with Oxone to give **4**. The radiochemical precursor **5** was generated by tosylation of **4** as described previously (Bertrand et al. 2016b). The non-radioactive standard was synthesized by fluorination of **4** using Deoxo-Fluor, followed by base-mediated ester hydrolysis (Bertrand et al. 2016b).

The radiosynthesis of **[**^**18**^**F]7** was completed with a nucleophilic radio-fluorination reaction followed by base-mediated ester hydrolysis in a one-pot, two-step process. Semi-preparative HPLC purification of **[**^**18**^**F]7** was initially explored using a C18 column. Co-elution of the radiotracer with a non-radioactive by-product was observed. Despite efforts, chromatographic resolution of the tracer from this by-product was not achieved by adjusting the mobile phase composition. High resolution mass spectrometry indicated that the identity of the impurity was the alkene-containing elimination by-product **8** (Fig. 1). Resolution of **[**^**18**^**F]7** from **8** was achieved employing a pentafluorophenyl-functionalized reverse phase column which enabled preparation of **[**^**18**^**F]7** in high purity. Inspection of previously described structure-activity relationships and the TAK-875/FFA-1 co-crystal structure predicted that **8** would potentially compete with **[**^**18**^**F]7** for binding to FFA-1 (Negoro et al. 2012; Srivastava et al. 2014). Thus, removal of **8** will increase the apparent molar activity of the final product and improve the potential for quantitative PET imaging of pancreatic FFA-1 and *β*-cell mass.

Reformulation of **[**^**18**^**F]7** was performed using an anion exchange resin. The purified **[**^**18**^**F]7** HPLC peak was collected into a vessel containing aqueous base to ensure ionization of the carboxylic acid functional group. From this solution, the carboxylate form of **[**^**18**^**F]7** was efficiently captured by the resin. The residual HPLC solvent was removed by washing with water. The radiotracer was then eluted from the resin with acidified ethanol, which was filtered and diluted into an isotonic bicarbonate and saline solution. This efficiently buffered the pH of the final injectable solution and provided **[**^**18**^**F]7** as a concentrated, injectable solution for animal PET studies.

Dynamic microPET/CT imaging (with or without a saturating dose of TAK-875) was conducted in rats to evaluate tracer kinetics and the potential of **[**^**18**^**F]7** as an FFA-1-targeting agent. The images displayed high uptake of the tracer in abdominal organs. While tracer uptake could be detected in the pancreas ROI, there was no reduction in tracer retention in the TAK-875 pre-treatment group. This suggests that the signal from the pancreas is confounded by non-specific binding in the pancreatic tissue. To further understand the in vivo value of this tracer and confirm these results, the focus of investigation was shifted to the analysis of tissue samples ex vivo.

Ex vivo biodistribution studies have the added benefit of eliminating error introduced when drawing the microPET/CT pancreatic ROI, which is indistinguishable from surrounding organs and tissues in rodents by CT and could only be estimated based on known anatomy (Yin et al. 2015). Theses studies indicated very little uptake of the radiotracer in the fat, bone, and muscle tissues. **[**^**18**^**F]7** was detected in the pancreas (0.54 ± 0.04 tissue-to-blood ratio). However, saturating FFA-1 by pre-treatment with TAK-875 did not lead to a decrease in tracer accumulation in the pancreas, which corroborated the microPET results. This led to an examination of the formation and presence of potential radiolabeled metabolites to characterize the radioactive signal that accumulated in the pancreas. Interestingly, HPLC radio-metabolite analysis exhibited only the presence of unchanged **[**^**18**^**F]7** in the pancreas at 60-min post-injection. The formation of one minor metabolite was also detected in the plasma. Together with the absence of radioactivity accumulation in the bone in the biodistribution studies, this result confirmed the relatively high metabolic stability of **[**^**18**^**F]7** in rats.

During the course of this work, a study found that [^3^H]TAK-875 exhibits a high level of off-target binding, likely related to the high degree of lipophilicity of the molecule (cLogP = 4.2) (Hellström-Lindahl et al. 2017). Given the similarity of **[**^**18**^**F]7** with TAK-875 in terms of chemical structure and lipophilicity (cLogP = 4.58), and with the results described here, it can be concluded that non-specific binding of **[**^**18**^**F]7** in the pancreatic tissue invalidates this tracer as an in vivo or ex vivo quantitative FFA-1 imaging agent. Future development of TAK-875-derived FFA-1 targeting radiotracers should be approached with a focus on decreasing off-target labeling in the pancreas and surrounding tissue by increasing the hydrophilicity.

## Conclusions

A one-pot, two-step fully automated process for the preparation of **[**^**18**^**F]7** was developed. The use of a pentafluorophenyl-functionalized HPLC column in this process allowed for the removal of the potential FFA-1 competitive impurity **8** and improved the purity and apparent molar activity of the final product. A strong anion-exchange resin was used to conduct rapid and efficient reformulation of the radiotracer in high concentration, enabling in vivo evaluation of **[**^**18**^**F]7** as a quantitative PET agent. MicroPET/CT imaging and biodistribution results exhibited activity accumulation in the pancreas although no specific binding could be detected following saturation studies of FFA-1 receptor. Radio-metabolite analysis indicated that **[**^**18**^**F]7** is sufficiently stable, ruling out the possibility that degradation was a contributing factor to the high degree of non-specific binding observed. While these results allow for the conclusion that non-specific binding of **[**^**18**^**F]7** in the pancreas impedes the ability to measure specific binding, they are instructive and provide direction for the development of future PET tracers for FFA-1-based *β*-cell PET imaging.

## Abbreviations

AM: Accurate mass
CT: Computed tomography
EM: Expectation maximization
EOS: End of synthesis
ESI: Electrospray ionization
FFA: Free-fatty acid receptor
HPLC: High performance liquid chromatography
HR: High resolution
ID: Injected dose
NMR: Nuclear magnetic resonance
OSEM: Ordered subset expectation maximization
PET: Positron emission tomography
RCY: Radiochemical yield
ROI: Region of interest
SD: Standard deviation
SPECT: Single photon emission computed tomography
SUV: Standardized uptake value
T2DM: Type II diabetes mellitus
THF: Tetrahydrofuran
